# Intratumor tertiary lymphatic structure evaluation predicts the prognosis and immunotherapy response of patients with colorectal cancer

**DOI:** 10.3389/fimmu.2024.1302903

**Published:** 2024-03-01

**Authors:** Huijing Feng, Siyuan Zhang, Qiuru Zhou, Fei Han, Gang Du, Lin Wang, Xuena Yang, Xiying Zhang, Wenwen Yu, Feng Wei, Xishan Hao, Xiubao Ren, Hua Zhao

**Affiliations:** ^1^ Department of Thoracic Oncology, Cancer Center, Shanxi Bethune Hospital, Shanxi Academy of Medical Sciences, Tongji Shanxi Hospital, Third Hospital of Shanxi Medical University, Taiyuan, Shanxi, China; ^2^ National Clinical Research Center for Cancer, Tianjin, China; ^3^ Key Laboratory of Cancer Prevention and Therapy, Tianjin, China; ^4^ Tianjin’s Clinical Research Center for Cancer, Tianjin, China; ^5^ Key Laboratory of Cancer Immunology and Biotherapy, Tianjin, China; ^6^ Department of Immunology, Tianjin Medical University Cancer Institute and Hospital, Tianjin, China; ^7^ Wenzhou Central Hospital, Wenzhou, Zhejiang, China; ^8^ Department of head and neck surgery, Shanxi Province Cancer Hospital/Shanxi Hospital Affiliated to Cancer Hospital, Chinese Academy of Medical Sciences/Cancer Hospital Affiliated to Shanxi Medical University, Taiyuan, Shanxi, China; ^9^ Department of Pathology, Shanxi Bethune Hospital, Taiyuan, Shanxi, China; ^10^ General Surgery Department, Shanxi Bethune Hospital, Taiyuan, Shanxi, China; ^11^ Haihe Laboratory of Cell Ecosystem, Tianjin, China; ^12^ Department of Biotherapy, Tianjin Medical University Cancer Institute and Hospital, Tianjin, China

**Keywords:** TLS, CRC, dMMR, pMMR, anti-PD1 immunotherapy

## Abstract

**Background:**

Immune checkpoint therapy, involving the programmed cell death 1 (PD-1) monoclonal antibody, has revolutionized the treatment of cancer. Tertiary lymphatic structure (TLS) serves as an immune indicator to predict the efficacy of PD-1 antibody therapy. However, there is no clear result whether the distribution, quantity, and maturity of TLS can be effective indicators for predicting the clinical efficacy of anti-PD1 immunotherapy in patients with colorectal cancer (CRC).

**Methods:**

Fifty-seven patients who underwent surgical resection and thirty-nine patients who received anti-PD-1 immunotherapy were enrolled in this retrospective study. Immunohistochemical staining and multiple fluorescence immunohistochemistry were used to evaluate the mismatch repair (MMR) subtypes and TLS distribution, quantity, and maturity, respectively.

**Results:**

A comprehensive patient score system was built based on TLS quantity and maturity. We found that the proportion of patients with score >1 was much higher in the deficient mismatch repair(dMMR) group than in the proficient mismatch repair(pMMR) group, and this difference was mainly due to intratumoral TLS. Patient score, based on the TLS evaluation of whole tumor, peritumor, or intratumor, was used to evaluate the efficacy of anti-PD1 immunotherapy. Based only on the intratumor TLS evaluation, the proportion of patients with a score >1 was higher in the response (PR + CR) group than in the non-response (PD) group. Multivariate analysis revealed that patient scores were positively correlated with the clinical efficacy of immunotherapy. Further analysis of immune-related progression-free survival was performed in patients with CRC who received anti-PD-1 immunotherapy. Patients with score >1 based on the intratumor TLS evaluation had significantly better survival.

**Conclusions:**

These results suggest that the patient score based on intratumor TLS evaluation may be a good immune predictive indicator for PD-1 antibody therapy in patients with CRC.

## 1 Introduction

Colorectal cancer (CRC) is a malignant tumor of the colon or rectum that is characterized by poor prognosis and high metastasis (1). According to global cancer statistics, CRC ranked third and second in terms of cancer incidence and mortality, respectively, in 2020 (2). Early CRC can be treated with radical surgical resection; however, surgery and adjuvant chemotherapy are not ideal for the treatment of advanced CRC (1). In recent years, novel immune checkpoint inhibitors (ICIs) have emerged as key therapeutics for patients with metastatic CRC with mismatch repair-deficient (dMMR) and -proficient (pMMR) subtypes according to MMR gene status (3). Although programmed cell death 1 (PD-1) inhibitors have been approved as the first-line treatment for advanced CRC with dMMR as a predictive biomarker for PD-1 ICIs, less than half of the patients with dMMR CRC respond favorably to anti-PD-1 therapy (3). Therefore, exploring the much accurate immune predictive indicators for PD-1 antibody therapy is necessary to guide treatment more accurately in patients with CRC with different MMR subtypes.

Tertiary lymphoid structures (TLSs), also known as ectopic lymphoid organs, develop in non-lymphoid tissues at sites of chronic inflammation, including tumors (4). Tumor-infiltrating lymphocytes, which may originate from the aggregation of ectopic immune cells at the tumor site, have drawn considerable interest because they play an important role in improving anti-tumor immunity (5, 6). Increasing evidence indicates that the presence and maturity of TLSs are correlated with tumor prognosis and can serve as novel prognostic biomarkers (7–10). Furthermore, TLSs can predict the responses to anti-PD‐1 immunotherapy and might be a target of PD‐1 blockade in several tumors including esophageal carcinoma, bladder cancer, melanoma and head and neck squamous cell carcinoma (HNSCC) (7–9, 11). It has also been demonstrated that high PD-1 expression in the invasive margin of patients was significantly associated with the presence of TLSs, which implies that targeting PD-1 in the immune context might be more effective (12). A recent study in mouse models of spontaneous multi-organ metastasis in MSI-H CRC tumors showed that ICIs of anti-PD-1 treatment significantly reduced the growth of primary tumors and liver metastases, and therapy efficacy correlated with the formation of TLSs in ICI-responding tumors. However, the utility of TLSs as predictive biomarkers for anti-PD-1 treatment of CRC remains unclear (13).

Efficacy of tumor immunotherapy is closely related to the MMR genotype. Greco et al. reported that patients with dMMR CRC have higher objective response rates and longer progression-free survival (PFS) after receiving immunotherapy than patients with pMMR (14). A recent study reported that patients with dMMR bladder cancer with increased tumor-resident memory T cells (T_RM_) infiltration contributing to TLS formation had improved response rates to neoadjuvant chemotherapy (15). However, the relationship between MMR status and TLS remain unclear.

In this study, we aimed to assess the correlation between MMR status and TLSs in CRC and explore TLSs as predictive biomarkers for anti-PD-1 immunotherapy to facilitate more personalized treatment of patients with CRC with different MMR subtypes.

## 2 Materials and methods

### 2.1 Patients and tumor specimens

Fifty-seven patients with CRC who underwent surgical resection between 2016 and 2019 at the Tianjin Medical University Cancer Hospital were enrolled in this retrospective study. Pathological TNM staging was based on the 8^th^ edition of the Union for International Cancer Control TNM classification. Formalin-fixed paraffin-embedded tumor tissue samples of these patients were collected for subsequent multiple immunofluorescence staining, in which 19 patients were dMMR positively expressing mismatch repair proteins, such as MLH1, PMS2, MSH2, and MSH6, and the 38 patients had pMMR-matched basic clinicopathological features with the former. None of the patients had received any therapy before surgery. Thirty-nine patients, comprising 10 dMMR patients and 29 pMMR patients, who received anti-PD1 immunotherapy between 2015 and 2021 at the Shanxi Provincial Cancer Hospital were enrolled in this study. None of the 39 patients ever underwent surgery or other treatments before pathological puncture biopsy.

Specimens from all patients were approved by the Ethics Committee of the Tianjin Medical University Cancer Institute and Hospital (Ek2020214) and the Shanxi Provincial Cancer Hospital (SBQLL-2022-028).

### 2.2 Multiple immunofluorescence staining and TLS quantification and scoring

A PerkinElmer Opal 7-color Technology Kit (NEL81001KT) was used to conduct immunofluorescence staining according to the manufacturer’s protocol. Antibodies against CD20 (1:800, ab9475, Abcam), CD21 (1:800, ab75985, Abcam), CK (1:800, ab215838, Abcam), BCL-6 (1:100, NBP3-07540, NOVUS), and GP2(1:400, D277-3, MBL) were used. Then, 4’,6-diamidino-2-phenylindole was used to stain the nuclei after completing all the staining cycles. An Automated Quantitative Pathology Imaging System (Vectra Polaris) was used to scan and visualize the stained slides, and the inForm image analysis software (v2.4.4; PerkinElmer) was used for quantification and scoring.

The method of evaluating TLS quantity and maturity was as follow:

Firstly, according to HE staining results, wax blocks with both normal and tumor tissues were selected to slice. Secondly, mIHC was performed to determinate the number and maturity of TLS.

We collected all TLSs of every tumor section and randomly collected three to five fields from areas outside the TLSs. The early TLS (Grade 1 TLS) was characterized by dense lymphocytic aggregates without CD21 and Bcl-6 expression; primary follicle-like TLS(Grade 2 TLS) was characterized by lymphocytic clusters with central network CD21 expression, but no GC reaction (Bcl-6-); and secondary follicle-like TLS (Grade 3 TLS) was characterized by lymphocytic clusters with GC reaction (CD20+Bcl-6+).

### 2.3 Statistical analyses

Statistical analyses were performed using SPSS 23.0 and GraphPad Prism (v.9.0). Shapiro Wilktest was used to test the normality of continuous variables, and the data normal distributing was described by mean ± standard deviation; data not subject to normal distribution was described by median and quartile. Comparisons of unpaired numerical variables between the two groups were assessed using Student’s *t*-test or Mann–Whitney test. X2 tests or Fisher’s exact test were used for comparisons between groups. Statistical significance was set at *P* value <0.05. When comparing the prognostic differences between the two subgroups after combining TLS quantity and maturity, *P* value and HR ratio were calculated using the log-rank test in GraphPad Prism software.

## 3 Results

### 3.1 Associations between CRC MMR state and TLS quantity and maturity

**Figure 1:**
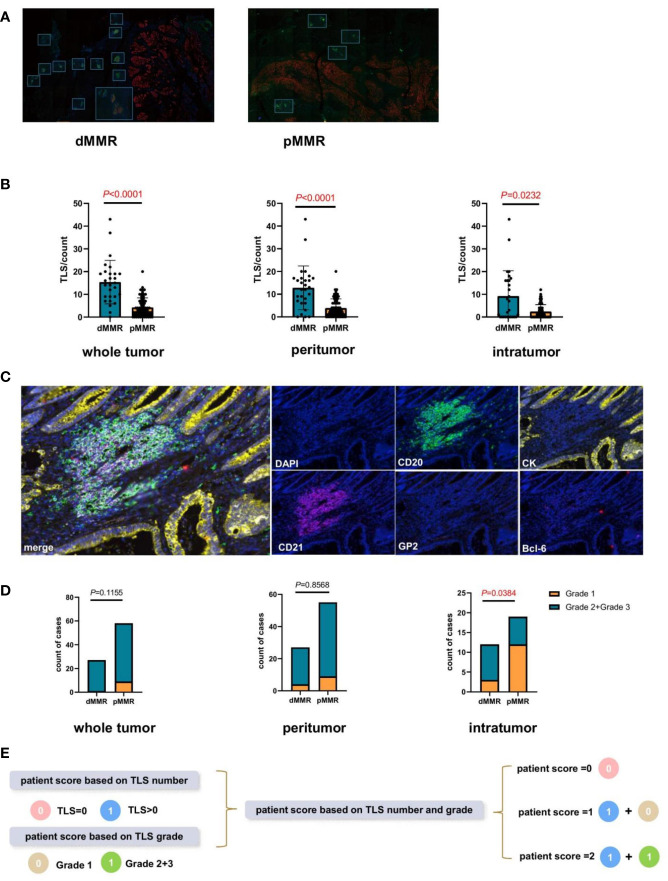
Comparison of the tertiary lymphatic structure (TLS) quantity and maturity between patients with dMMR and pMMR CRC (n=96). **(A)** Representative images of TLS number of dMMR and pMMR patient samples (magnification, ×100). The slide was stained with CK, CD21 (red), CD20 (green), Bcl-6 (red), GP2 (orange), and 4’,6-diamidino-2-phenylindole (DAPI; blue). **(B)** Comparison of TLS number between dMMR and pMMR patients in three levels: whole tumor (*P*<0.0001), peritumor (*P*<0.0001), and intratumor (*P*<0.0001) levels. **(C)** Representative images of TLS maturity (magnification, ×100). The slide was stained with GP2 (orange), CD21 (purple), CD20 (green), CK (yellow), Bcl-6 (pink), and DAPI (blue). Grade1-TLS, both CD21 and Bcl-6 markers were negative and GP2 was negative. Grade2-TLS, CD21 was positive and Bcl-6 and GP2 were negative. Grade3-TLS, both CD21 and Bcl-6 markers were positive and GP2 was negative. **(D)** Comparison of the patient proportion between dMMR and pMMR patients based on the TLS grade in the whole tumor (*P*=0.1155), peritumor (*P*=0.8568), and intratumor (*P*<0.05) groups, respectively. **(E)** Establishment of the new patient score system integrating TLS quantity and maturity grade.

To explore the TLS difference between dMMR and pMMR, 96 mIHC staining samples of patients with CRC were analyzed (**Table 1**). TLS with CD20^+^CD21^-^BCL6^-^ was defined as grade 1, TLS with CD20^+^CD21^+^BCL6^-^ was defined as grade 2, and TLS with CD20^+^CD21^+^BCL6^+^ was defined as grade 3 (16). In addition, the GP2^+^ lymphoid tissue represented a payer patch that was excluded, and CK was used to differentiate the intratumoral and peritumoral regions (**Figure 1C**). We found that, regardless of the level of intratumor, peritumor, or whole sample, TLS quantity was higher in dMMR than in pMMR (*P*<0.0001, *P*<0.0001, *P*<0.0001; **Figures 1A, B**), while TLS maturity was higher in dMMR than in pMMR only at the intratumor level (*P*<0,05; **Figures 1C, D**).

**Table 1:** Baseline characteristics of patients (n=96).

		dMMR, n=28 (%)	pMMR, n=68 (%)	*P* value
Age				0.385
	<65	24 (86.0)	53 (78.0)	
	≥65	4 (14.0)	15 (22.0)	
Gender				0.095
	Male	20 (71.0)	36 (53.0)	
	Female	8 (29.0)	32 (47.0)	
Tumor location				0.202
	Right hemicolon	18 (64.0)	34 (50.0)	
	Left hemicolon	10 (36.0)	34 (50.0)	
T stage				0.394
	T1+T2	2 (8.0)	9 (15.0)	
	T3+T4	23 (92.0)	52 (85.0)	
N stage				0.777
	N0	15 (60.0)	34 (57.0)	
	N1+N2	10 (40.0)	26 (43.0)	
TNM stage				0.855
	I	2 (7.0)	7 (10.0)	
	II	12 (44.0)	24 (36.0)	
	III	10 (37.0)	26 (39.0)	
	IV	3 (12.0)	10 (15.0)	

Therefore, a comprehensive patient scoring system was built based on the TLS quantity and maturity. Scores based on the TLS number were defined as 0 (TLS number=0) or 1(TLS number >0). The score based on TLS maturity was defined as 0 (TLS grade 1) or 1 (TLS grade 2 or 3). Based on the sum of these two scores, the patient scores were calculated and divided into 0, 1, and 2 (**Figure 1E**).

### 3.2 Comparison of patient score differences between dMMR and pMMR patients

**Figure 2:**
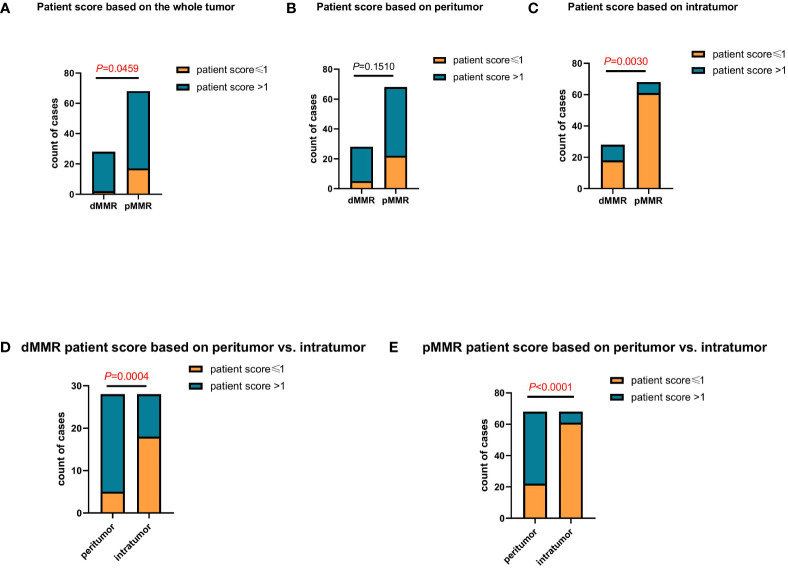
Comparing the differences in patient scores between patients with dMMR and pMMR CRC (n=96). **(A–C)** Comparison of patient score between patients with dMMR and pMMR CRC at the whole tumor (*P*<0.05) **(A)**, peritumor (*P*=0.1510) **(B)**, and intratumor (*P*<0.005) **(C)** levels. **(D, E)** Comparison of patient score at the peritumor and intratumor levels between patients with dMMR (*P*<0.005) **(D)** and pMMR (*P*<0.0001) **(E)** CRC, respectively.

The proportions of patients with different scores based on the peritumor, intratumor, and whole tumor microenvironment were analyzed separately. Based on the whole tumor, the proportion of patients with a score >1 in the dMMR group was much higher than that in the pMMR group (*P*=0.0459; **Figure 2A**). Continuing the analysis, we found that this difference was mainly due to intratumoral TLS, but not peritumoral TLS (*P*=0.0459, *P*=0.1510; **Figures 2B, C**).

In addition, we compared the proportion of peritumor and intratumor patient scores between the dMMR and pMMR groups. In both the dMMR and pMMR groups, the proportion of patients with a score >1 based on the intratumor was much lower than that in the peritumor group (*P*=0.004, *P*<0.0001; **Figures 2D, E**). Since the proportion of patients scoring >1 based on intratumor TLS was very similar to the previously reported clinical response rate (17), it indicated that the patient score based on intratumor TLS might be an effective indicator in anti-PD1 immunotherapy.

### 3.3 Association between the anti-PD-1 response and patient score

**Figure 3:**
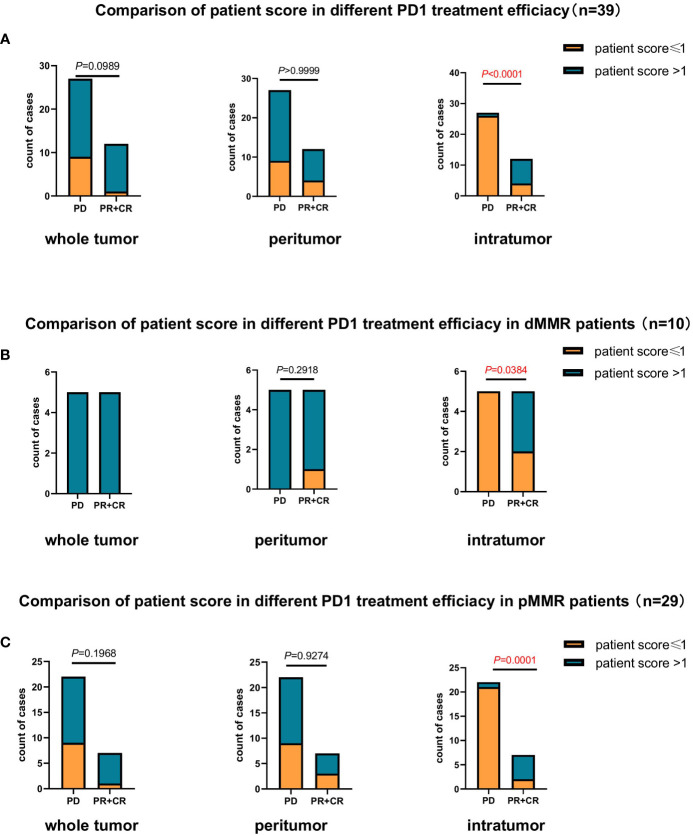
Comparison of the association between patient scores based on TLS quantity and maturity and response to anti-PD1 therapy (n=39). **(A)** Comparison of the association between patient score and response to anti-PD1 therapy in patients with CRC (n=39) at the whole tumor (*P*=0.0989), peritumor (*P*>0.9999), and intratumor (*P*<0.0001) levels. **(B)** Comparison of the association between patient score and response to anti-PD1 therapy in patients with dMMR CRC (n=10) at the whole tumor, peritumor (*P*=0.2918) and intratumor (*P*<0.05) levels. **(C)** Comparison of the association between patient score and response to anti-PD1 therapy in patients with pMMR CRC (n=29) at the whole tumor (*P*=0.1968), peritumor (*P*=0.9274), and intratumor (*P*=0.0001) levels.

In the present study, 39 patients with CRC received anti-PD-1 immunotherapy were enrolled to further validate the predictive role of the TLS score in the efficacy of PD-1 inhibitor therapy (**Table 2**). The patients were divided into two groups according to their therapeutic responses. Patients with a partial response (PR) and complete response (CR) were assigned to the response group, and those with progressive disease (PD) were assigned to the non-responsive group. The results showed that based on the intratumor TLS score, the proportion of patients with a score >1 in the PR+CR group was much higher than that in the PD group (*P*<0.0001; **Figure 3A**). Furthermore, we continued to evaluate the treatment response in patients with dMMR and pMMR based on TLS scores, and the data showed similar results to those of all patients. Based only on the intratumor TLS score, the proportion of patients with a score >1 was higher in the response group than in the non-response group (*P*=0.0384, *P*=0.0001; **Figures 3B, C**).

**Table 2:** Baseline characteristics of patients (n=39).

		dMMR, n=10 (%)	pMMR, n=29 (%)	*P* value
Age				0.086
	<65	10 (100.0)	22 (76.0)	
	≥65	0 (0.0)	7 (24.0)	
Gender				0.164
	Male	8 (80.0)	16 (55.0)	
	Female	2 (20.0)	13 (45.0)	
Tumor location				0.105
	Right hemicolon	6 (60.0)	9 (31.0)	
	Left hemicolon	4 (40.0)	20 (69.0)	
T stage				P>0.9999
	T1+T2	0 (0.0)	0 (0.0)	
	T3+T4	7 (100.0)	22 (100.0)	
N stage				0.483
	N0	3 (43.0)	6 (29.0)	
	N1+N2	4 (57.0)	15 (71.0)	
TNM stage				0.429
	II	2 (22.0)	3 (11.0)	
	III	4 (44.0)	15 (54.0)	
	IV	3 (33.0)	10 (35.0)	
In combination with chemotherapy				
	yes	4 (40%)	23 (79%)	0.0202
	no	6 (60%)	6 (21%)	

Multiple clinical, pathological, and immune characteristics were investigated to evaluate their impact on the clinical response to anti-PD1 immunotherapy. The results revealed that Patient scores were positively correlated with clinical efficacy in the 39 patients and pMMR group (*P*=0.004, *P*=0.012, **Tables 3**, **4**). Multivariate analysis was not performed in the dMMR group because of the small number of enrolled patients.

**Table 3:** Multivariate clinical pathological characteristics and immune characteristics affecting response of anti-PD1 immunotherapy in 39 pMMR patients.

	B	S.E.	Wald	df	Sig.	Exp (B)	95% C.I.for EXP (B)	
							Lower	Upper
TLS score	5.153	1.799	8.207	1	0.004	172.872	5.091	5869.952
Gender	2.269	1.586	2.048	1	0.152	9.669	0.432	216.298
Age	2.521	1.921	1.722	1	0.189	12.447	0.288	537.535
Location	1.294	1.384	0.874	1	0.35	3.646	0.242	54.931
Therapy	-0.967	1.536	0.396	1	0.529	0.38	0.019	7.728
TNM stage	-0.105	0.794	0.017	1	0.895	0.901	0.19	4.273
dMMR/pMMR	-1.413	1.432	0.974	1	0.324	0.244	0.015	4.028

**Table 4:** Multivariate clinical pathological characteristics and immune characteristics affecting response of anti-PD1 immunotherapy in 29 pMMR patients.

	B	S.E.	Wald	df	Sig.	Exp (B)	95% C.I.for EXP (B)	
							Lower	Upper
TLS score	4.259	1.690	6.351	1	0.012	70.741	2.577	1941.971
Gender	0.930	1.609	0.334	1	0.563	2.535	0.108	59.393
Age	1.405	1.831	0.589	1	0.443	4.076	0.113	147.406
Location	-0.494	1.743	0.080	1	0.777	0.610	0.020	18.599
Therapy	-0.167	2.173	0.006	1	0.939	0.846	0.012	59.832
TNM stage	-0.506	1.035	0.239	1	0.625	0.603	0.079	4.585

### 3.4 Predictive role of patient score in determining the immune-related PFS in anti-PD1 therapy

**Figure 4:**
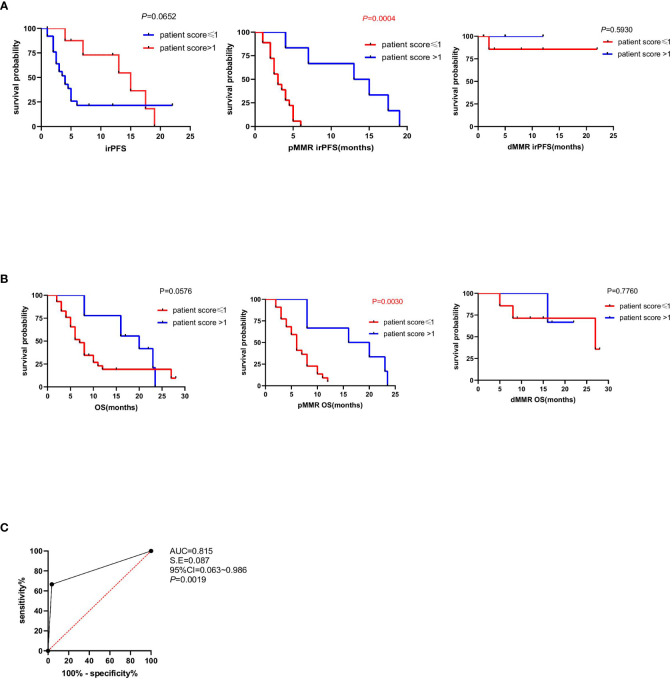
Association between patient score and immune-related progression-free survival (irPFS and OS). **(A)** Kaplan–Meier survival curves showing irPFS according to patient score of all the 39 patients, pMMR patients and dMMR patients (*P*=0.0652, *P*=0.0004, *P*=0.5930). *P* values were calculated by the log-rank test. **(B)** Kaplan–Meier survival curves showing OS according to patient score of all the 39 patients, pMMR patients and dMMR patients. *P* values were calculated by the log-rank test (*P*=0.0576, *P*=0.0030, *P*=0.7760). **(C)** The ROC curve to evaluate the predictive ability of patient score on anti-PD1 immunotherapy clinical responses (AUC=0.815).

To further investigate the patient score on clinical efficacy prediction, survival analysis of irPFS and multivariate Cox regression analyses of clinical and immune characteristics in different patient score group was done, which verified that the irPFS of patients score>1 was longer than that of patients score ≤ 1 in pMMR group (*P*=0.0004; **Figure 4A**; **Table 5**). The irPFS in dMMR group did not show any difference, which may have been due to its small sample size (n=10, **Figure 4A**). Survival analysis of OS was also done, which verified that the OS of patients score>1 was longer than that of patients score ≤ 1 in pMMR group but not the whole 39 patients and dMMR group (*P*=0.0030, *P*=0.0576 and *P*=0.7760, respectively, **Figure 4B**). In addition, receiver operating characteristic (ROC) curve analysis was performed in pMMR patients, revealing that the patient score had good predictive ability for the clinical efficacy of PD1 treatment (AUC=0.815; **Figure 4C**). All the results indicated that the patient score based on the intratumor microenvironment can be used as a predictive factor for anti-PD1 immunotherapy in patients with CRC.

**Table 5:** Multivariate Cox regression analyses of clinical and immune characteristics affecting irPFS of anti-PD1 immunotherapy in 29 pMMR patients.

	B	SE	Wald	df	Sig.	Exp (B)	95.0% CI for Exp (B)	
							Lower	Upper
TLS score	-3.337	1.171	8.127	1	0.004	0.036	0.004	0.352
Gender	-0.198	0.448	0.194	1	0.660	0.821	0.341	1.976
Age	-0.253	0.576	0.193	1	0.660	0.776	0.251	2.400
Location	-0.361	0.575	0.393	1	0.530	0.697	0.226	2.152
Therapy	-1.368	0.729	3.517	1	0.061	0.255	0.061	1.064
TNM stage	0.133	0.375	0.126	1	0.723	1.142	0.548	2.381

## 4 Discussion

In this study, we first evaluated the associations between MMR typing and TLS distribution, quantity, and maturity, clinical features, and prognosis of 96 patients with stage I–IV CRC. In the analysis of TLS quantity difference of the whole tumor, peritumor, or intratumor microenvironment between patients with dMMR and pMMR CRC, the results showed that the number of TLS was much higher in dMMR than in pMMR patients, which was consistent with the results of a study on the immunomicroenvironment characteristics of urachal carcinoma (UrC). They found that the number of TLS tended to be higher in UrC tumor with dMMR (*P*=0.1919), as well the patients with higher TLS numbers tended to result in a much better prognosis (18). TLS maturity analysis revealed no differences in the whole tumor or peritumor between the dMMR and pMMR groups. In the intratumor-TLS analysis, the proportion of patients with grade 2+grade 3 pMMR was lower than that of patients with dMMR. The maturity of intratumoral TLS was higher in patients with dMMR than in those with pMMR. This is consistent with the findings of CRC and lung squamous cell carcinoma, in which patients from germinal centers (GCs) had a better prognosis (19, 20). These results further indicated that B cell maturity and humoral immunity play key roles in anti-tumor immune responses (16).

Based on the contribution of TLS quantity and maturity to the anti-tumor response, we used a patient scoring system (PS) to predict the clinical response of patients with CRC to PD-1 antibodies immunotherapy. Using this new score, we analyzed the relationship between TLS and MMR subtype and the results showed both in dMMR and pMMR patients, the proportion of patients score >1 based on intratumor was much lower than that peritumor. Moreover, in either the dMMR group or the pMMR group, the proportion of patients scoring >1 based on intratumoral TLS was similar to the clinical response rate that has been reported (17), and whether the patient score based on intratumoral TLS could be an effective indicator of anti-PD1 immunotherapy.

In this study, we analyzed the correlation between patient scores and clinical responses in 39 patients receiving anti-PD-1 immunotherapy and found that the proportion of patients with intratumoral TLS patient scores > 1 was much higher in the response (PR + CR) group than in the non-responders groups. Similar results were observed in 29 pMMR and 10 dMMR patients. These results suggest that the distribution of TLS affects the efficacy of immunotherapy. Previous studies defined the peritumoral TLS of breast cancer as a range within 5 mm from the invasive edge and divided it into adjacent and distal TLS according to the distance and interval between the normal breast tissue and the invasive edge. The higher the peritumoral TLS (distal TLS) density, the lower is the disease-free survival (DFS), independent of overall survival (OS), and the higher the distal TLS density, the lower is the DFS and OS (21). This is consistent with the results of the present study; however, no similar studies have been reported in CRC to date.

Further analysis of irPFS was performed in 39 patients with CRC who received anti-PD-1 immunotherapy. We found that patients with score > 1 based on the intratumor TLS evaluation had much better survival among the 29 patients with pMMR. Although the same trend was observed in the dMMR group, statistical results are not available because of the small number of cases. The specimens of the 39 patients receiving anti-PD-1 treatment were collected before immunotherapy and the sample size was limited, which might lead to a potential bias. In future research, we will collect more specimens and conduct prospective studies to verify the authenticity and accuracy of the conclusions. In conclusion, our findings indicate the patient score based on intratumor TLS evaluation as a good immune predictive indicator for the efficacy of PD-1 antibody therapy in patients with CRC.

## Data availability statement

The original contributions presented in the study are included in the article/supplementary material. Further inquiries can be directed to the corresponding authors.

## Ethics statement

The studies involving humans were approved by the Ethics Committee of the Tianjin Medical University Cancer Institute and Hospital and the Ethics Committee of Shanxi Provincial Cancer Hospital. The studies were conducted in accordance with the local legislation and institutional requirements. The human samples used in this study were acquired from primarily isolated as part of your previous study for which ethical approval was obtained. Written informed consent for participation from the participants or the participants’ legal guardians/next of kin was not mentioned in accordance with the national legislation and institutional requirements.

## Author contributions

HF: Formal analysis, Methodology, Writing – original draft. SZ: Formal analysis, Methodology, Writing – original draft. QZ: Writing – review & editing. FH: Writing – review & editing. GD: Writing – review & editing. LW: Writing – review & editing. XY: Writing – review & editing. XZ: Writing – review & editing. WY: Writing – review & editing. FW: Writing – review & editing. XH: Writing – review & editing. XR: Project administration, Writing – review & editing. HZ: Project administration, Writing – review & editing.
