# Corrigendum: Intratumor tertiary lymphatic structure evaluation predicts the prognosis and immunotherapy response of patients with colorectal cancer

**DOI:** 10.3389/fimmu.2024.1397764

**Published:** 2024-06-14

**Authors:** Huijing Feng, Siyuan Zhang, Qiuru Zhou, Fei Han, Gang Du, Lin Wang, Xuena Yang, Xiying Zhang, Wenwen Yu, Feng Wei, Xishan Hao, Xiubao Ren, Hua Zhao

**Affiliations:** ^1^ Department of Thoracic Oncology, Cancer Center, Shanxi Bethune Hospital, Shanxi Academy of Medical Sciences, Tongji Shanxi Hospital, Third Hospital of Shanxi Medical University, Taiyuan, Shanxi, China; ^2^ National Clinical Research Center for Cancer, Tianjin, China; ^3^ Key Laboratory of Cancer Prevention and Therapy, Tianjin, China; ^4^ Tianjin’s Clinical Research Center for Cancer, Tianjin, China; ^5^ Key Laboratory of Cancer Immunology and Biotherapy, Tianjin, China; ^6^ Department of Immunology, Tianjin Medical University Cancer Institute and Hospital, Tianjin, China; ^7^ Wenzhou Central Hospital, Wenzhou, Zhejiang, China; ^8^ Department of head and neck surgery, Shanxi Province Cancer Hospital/Shanxi Hospital Affiliated to Cancer Hospital, Chinese Academy of Medical Sciences/Cancer Hospital Affiliated to Shanxi Medical University, Taiyuan, Shanxi, China; ^9^ Department of Pathology, Shanxi Bethune Hospital, Taiyuan, Shanxi, China; ^10^ General Surgery Department, Shanxi Bethune Hospital, Taiyuan, Shanxi, China; ^11^ Haihe Laboratory of Cell Ecosystem, Tianjin, China; ^12^ Department of Biotherapy, Tianjin Medical University Cancer Institute and Hospital, Tianjin, China

**Keywords:** TLS, CRC, dMMR, PMMR, anti-PD1 immunotherapy

In the published article, there was an error in affiliation(s) 2, 3, 4, 5, 6, 7, 8, 9, 10, 11, 12, 13. Instead of “^2^Tianjin Medical University Cancer Institute and Hospital, Tianjin China, ^3^National Clinical Research Center for Cancer, Tianjin, China, ^4^Key Laboratory of Cancer Prevention and Therapy, Tianjin China, ^5^Tianjin’s Clinical Research Center for Cancer, Tianjin China, ^6^Key Laboratory of Cancer Immunology and Biotherapy, Tianjin, China, ^7^Department of Immunology, Tianjin Medical University Cancer Institute and Hospital, Tianjin, China, ^8^Wenzhou Central Hospital, Wenzhou, Zhejiang, China, ^9^Department of Head and Neck Surgery, Shanxi Province Cancer Hospital/ Shanxi Hospital Affiliated to Cancer Hospital, Chinese Academy of Medical Sciences/Cancer Hospital Affiliated to Shanxi Medical University, Taiyuan, Shanxi, China, ^10^Department of Pathology, Shanxi Bethune Hospital, Taiyuan, Shanxi, China, ^11^General Surgery Department, Shanxi Bethune Hospital, Taiyuan, Shanxi, China, ^12^Haihe Laboratory of Cell Ecosystem, Tianjin, China, ^13^Department of Biotherapy, Tianjin Medical University Cancer Institute and Hospital, Tianjin, China.”, it should be:” ^2^National Clinical Research Center for Cancer, Tianjin, China, ^3^Key Laboratory of Cancer Prevention and Therapy, Tianjin, China, ^4^Tianjin’s Clinical Research Center for Cancer, Tianjin, China, ^5^Key Laboratory of Cancer Immunology and Biotherapy, Tianjin, China, ^6^Department of Immunology, Tianjin Medical University Cancer Institute and Hospital, Tianjin, China, ^7^Wenzhou Central Hospital, Wenzhou, Zhejiang, China, ^8^Department of head and neck surgery, Shanxi Province Cancer Hospital/Shanxi Hospital Affiliated to Cancer Hospital, Chinese Academy of Medical Sciences/Cancer Hospital Affiliated to Shanxi Medical University, Taiyuan, Shanxi, China, ^9^Department of Pathology, Shanxi Bethune Hospital, Taiyuan, Shanxi, China, ^10^General Surgery Department, Shanxi Bethune Hospital, Taiyuan, Shanxi, China, ^11^Haihe Laboratory of Cell Ecosystem, Tianjin, China, ^12^Department of Biotherapy, Tianjin Medical University Cancer Institute and Hospital, Tianjin, China.”.

The authors apologize for this error and state that this does not change the scientific conclusions of the article in any way. The original article has been updated.

In the published article, there was an error regarding the affiliation(s) for Siyuan Zhang, Qiuru Zhou, Fei Han, Gang Du, Lin Wang, Xuena Yang, Xiying Zhang, Wenwen Yu, Feng Wei, Xishan Hao, Xiubao Ren and Hua Zhao.

Instead of Siyuan Zhang^2,3,4,5,6,7†^, Qiuru Zhou^2,3,4,5,6,7,8^, Fei Han^9^, Gang Du^10^, Lin Wang^11^, Xuena Yang^7^, Xiying Zhang^7^, Wenwen Yu^7^, Feng Wei^7,12^, Xishan Hao^12^, Xiubao Ren^7,12,13*^ and Hua Zhao^7,12*^, it should be: Siyuan Zhang^2,3,4,5,6†^, Qiuru Zhou^2,3,4,5,6,7^, Fei Han^8^, Gang Du^9^, Lin Wang^10^, Xuena Yang^2,3,4,5,6^, Xiying Zhang^2,3,4,5,6^, Wenwen Yu^2,3,4,5,6^, Feng Wei^2,3,4,5,6,11^, Xishan Hao^2,3,4,5,11^, Xiubao Ren^2,3,4,5,6,11,12*^ and Hua Zhao^2,3,4,5,6,11*^

